# The function of RNA-binding proteins at the synapse: implications for neurodegeneration

**DOI:** 10.1007/s00018-015-1943-x

**Published:** 2015-06-06

**Authors:** Chantelle F. Sephton, Gang Yu

**Affiliations:** Department of Psychiatry and Neuroscience, Institut Universitaire En Santé Mentale de Québec, Université Laval, Quebec, QC G1J 2G3 Canada; Department of Neuroscience, University of Texas Southwestern Medical Center, Dallas, TX 75390 USA

**Keywords:** Amyotrophic lateral sclerosis, Frontotemporal dementia, Local translation, RNA granules, RNP granules, Stress granules, FUS, TDP-43

## Abstract

The loss of synapses is a central event in neurodegenerative diseases. Synaptic proteins are often associated with disease neuropathology, but their role in synaptic loss is not fully understood. Of the many processes involved in sustaining the integrity of synapses, local protein translation can directly impact synaptic formation, communication, and maintenance. RNA-binding proteins and their association with RNA granules serve to regulate mRNA transportation and translation at synapses and in turn regulate the synapse. Genetic mutations in RNA-binding proteins FUS and TDP-43 have been linked with causing neurodegenerative diseases: amyotrophic lateral sclerosis and frontotemporal dementia. The observation that mutations in FUS and TDP-43 coincide with changes in RNA granules provides evidence that dysfunction of RNA metabolism may underlie the mechanism of synaptic loss in these diseases. However, we do not know how mutations in RNA-binding proteins would affect RNA granule dynamics and local translation, or if these alterations would cause neurodegeneration. Further investigation into this area will lead to important insights into how disruption of RNA metabolism and local translation at synapses can cause neurodegenerative diseases.

## Introduction

Translational control occurs mostly by homeostatic responses that alter general protein synthesis. However, gene-specific translational control depends on regulatory elements in the mRNA, such as upstream open reading frames, secondary structures or regulatory protein-binding sites [1]. As such, mRNA specificity in translational control can be achieved by the general translation machinery or by RNA-binding proteins, which are the main group of proteins that regulate mRNA transport and protein translation at synapses. In particular, RNA-binding proteins help meet the demands of the ever-changing microenvironment of the neuron which include responses to synaptic depolarization and depression, reduced nutrient availability, oxidative stress, misfolded proteins, and apoptosis. It is both a mystery and a marvel how large complexes of RNA-binding proteins and other core proteins coordinate mRNA transport and translation in response to these cues.

The importance of RNA-binding protein function at synapses is highlighted in patients with neurological and neurodegenerative disorders where genetic mutations or deletions of genes encoding RNA-binding proteins result in loss of synaptic plasticity and neuron function. Genetic mutations or deletions in disorders of autism, fragile X syndrome and Rett syndrome, which also correspond with a loss of synaptic plasticity and function, strongly suggest that disruption of RNA regulation is a central cause of synaptic defects in these brain disorders. Neurodegenerative disorders, such as amyotrophic lateral sclerosis (ALS) and frontotemporal dementia (FTD), are related by overlapping clinical phenotypes, genetic links and rapid disease progression [2]. Whereas ALS is a motor neuron disease and is caused by selective degeneration of motor neurons, which results in gradual muscle weakness and atrophy and death [3], it also has loss of synapses as part of the disease. FTD is a common form of dementia and is characterized by atrophy of the frontal and temporal lobes, which cause changes in behavior, cognition, and changes in personality and/or language [4]. FTD can be accompanied by loss of motor neuron function, and up to 75 % of ALS patients experience behavior and cognitive impairment [5]. This has led to the conclusion that the cause of ALS and FTD are somehow linked. With the advances in genetic screening, what has emerged is a strong association with several RNA regulatory proteins with causing familial forms of both ALS and FTD [6, 7]. Given this information altered RNA regulation is likely the underlying cause of these diseases and is at the forefront of understanding the mechanism behind ALS and FTD. This review will highlight the role of RNA-binding proteins in regulating local translation, their impact on maintaining synapses and the potential role of disease-linked RNA-binding proteins, Fused in sarcoma (FUS) and Transactive response DNA-binding protein (TDP-43) in the dysregulation of synaptic function and the initiation of neurodegeneration.

## The role of RNA granules at synapses

Neurons are highly complex cells with polarized and elaborate processes that extend long distances in the central nervous system. The distance between the synapse and cell body creates a supply and demand challenge for neurons, particularly at synapses. Neurons have the challenge of regulating the local translation of proteins at the synapse in order to meet the rapidly changing environment of neuronal inputs. The solution to these demands requires a local mechanism for controlling transport of mRNA to synapses and regulation of translation to allow local synthesis of new synaptic proteins at a moment’s notice. This is achieved by having all of the necessary components for translation; mRNA, ribosomes and translation factors, present in dendrites and even in the dendritic spines, ready for local protein synthesis [8, 9]. The regulation of local protein synthesis is particularly interesting because it allows neurons to rapidly modulate the production of proteins independent of new transcription or mRNA transport, which can modify the synapse directly. For instance, the localization of mRNAs at synapses and local protein synthesis is demonstrated to be critical for synaptic plasticity and the consolidation and storage of information in the brain [10]. Likewise, mRNA targeting and local protein synthesis have also been shown to influence axon guidance and nerve regeneration [11].

**Fig. 1:**
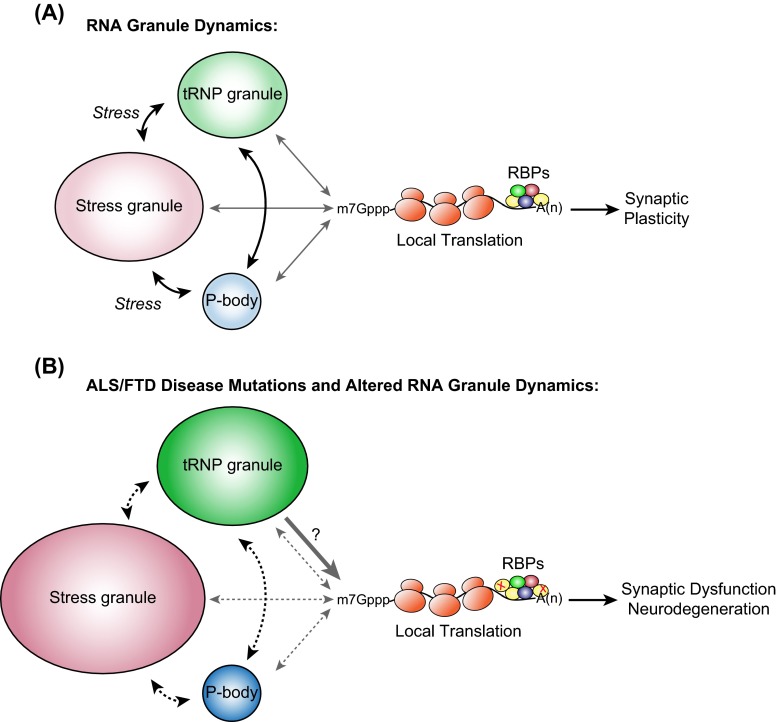
Model of RNA granule dynamics in neurodegenerative disorders. **a** RNA-binding proteins associate with RNAs to form mRNPs, which assemble into large, diverse multi-mRNP complexes like tRNPs, stress granules, or processing bodies. tRNP granules determine the cytoplasmic localization and fate of the mRNA and keep the mRNA in a translationally dormant state. tRNP granules can associate and exchange mRNPs with stress granules and processing bodies in response to cellular cues such as stress. mRNAs are protected within stress granules during times of stress and serve as sites of mRNA triage where mRNP complexes are monitored for integrity and composition and are then routed to sites of reinitiation, degradation or storage. Once the stress has been removed, stress granules disassemble, mRNAs are repacked into translationally competent mRNAs and proteins are synthesized or are selectively exported to associated processing bodies for degradation. Processing bodies are sites of mRNA degradation, mRNA surveillance, translational repression, RNA-mediated silencing, and may also be involved in storage of select RNAs and recycling/modification of decay factors. Processing bodies can associate with tRNPs, stress granules, and translation machinery. Throughout the different exchanges between mRNP:RNA granules and mRNP:translation machinery, RNA-binding proteins are associated with their target mRNAs. Following translation, mRNPs can assemble back into translationally repressed tRNP granules, degraded or assembled into processing bodies. For a functioning neuron, these dynamic exchanges are important factors in the quality control of local translation at synapses and the maintenance of synaptic communication and plasticity. **b** Depicted is a model of how ALS/FTD mutations in FUS and TDP-43 affect RNA granule dynamics and the impact on translation and synaptic function. FUS-disease mutations cause an increase in number and size of both tRNP and stress granules. The impact of this may be two-fold: (1) FUS mutations which cause more spontaneous assembly of tRNP granules and increased translational activities would impact the normal processes of the neuron; and (2) FUS mutations which cause tRNP and stress granules to be more insoluble would lead to “seeding” of insoluble pathological inclusions associated with ALS and FTD. However, the insoluble nature of both tRNP and stress granules could also impact translation in a negative manner, which has yet to be determined. Additionally, FUS-disease mutations negatively impact the formation of processing bodies and solubility of stress granules, which would likely alter the normal functions and of these RNA granules. TDP-43-disease mutations on the other hand cause larger and fewer tRNP granules in the dendrites as well as larger and more stress granules to form in response to stress. The consequences of this may be very similar to what occurs with FUS mutations including reduced RNA granule exchanges, altered translational activities and increased “seeding” of insoluble protein aggregates. There have been no changes observed with processing bodies, but the insolubility of stress granules would predict a disturbance in mRNP:RNA granule exchanges. The net impact of FUS- and TDP-43-disease mutations would be altered RNA granule dynamics, leading to misregulation of mRNA and translation, which would impact synaptic function and cause neurodegeneration. (*RBP* RNA-binding protein, *x* a mutation in an RNA-binding protein, *black arrows* RNA granule exchanges, *gray arrows* RNA granule interactions with translation machinery, *broken arrows* altered RNA granule dynamics)

Messenger ribonucleoprotein complexes (mRNPs) form when mRNA associates with protein complexes. In higher eukaryotes, mRNPs comprise more than ten thousand different RNA sequences and hundreds of different RNA-binding proteins. mRNPs can also assemble into more complex structures known as RNA granules (or RNP granules). There are many types of RNA granules, for example, transport RNP granules (tRNP), stress granules, processing bodies, germ granules, and nuclear paraspeckles [12]. The classification of RNA granules is based on their composition, subcellular localization, cell of origin, response to stimuli, dynamic behavior, and proposed functions [12, 13]. RNA granules can form in response to cellular inputs and environmental cues. In turn, they regulate the distribution, translation, and degradation of mRNA transcripts. RNA granules do not function as isolated particles, but instead constantly interact with each other, exchanging mRNPs, cytosolic proteins, and with polysomes (Fig. 1a) (reviewed in [14]). In essence, the formation of different granules regulates mRNA and protein synthesis, which directly impact the neuron’s fate. Common to all RNA granules is the presence of RNA-binding proteins, which associate with mRNA in untranslated regions (5′UTR or 3′UTR) or coding regions [15, 16] and are largely responsible for coordinating thier localization, stability, and translation. Transport RNP granules, stress granules and processing bodies have been linked with the pathology of a variety of diseases and we will focus our discussion on their role in maintaining neuronal function and how disruption of these granules may lead to disease.

Transport RNP granules (tRNP granule) are ribonucleoprotein particles that function in transport and storage of mRNA and can contain miRNA [17, 18]. Several core protein components of tRNP granules include RNA-binding proteins such as Staufen1, Staufen2, Fragile X mental retardation protein 1 (FMR1 commonly known as FMRP), heterogeneous nuclear ribonucleoprotein A2 (hnRNPA2), cytoplasmic polyadenylation element-binding protein (CPEB), survival of motor neuron protein (SMN), zipcode-binding protein 1 (ZBP1), and Purine-Rich Element-Binding Protein alpha (Purα), which participate in dendritic transcript transport [19]. Other RNA-binding proteins that are found in tRNP granules include, Smaug, Paumilio, FUS, and TDP-43 [20–24]. More than 40 different proteins including motor proteins (i.e., kinesin) have been identified in tRNP granules, many of which are proteins related to RNA transport and regulation, protein synthesis and several of which their function is unknown [23, 25, 26]. These tRNP protein components may or may not be “essential” for tRNP granule formation; however, depending on the context may be essential for regulating mRNA fate.

Stress granules are cytoplasmic aggregates composed of RNA binding proteins, RNA and stalled translation initiation complexes that usually form in a reversible manner upon cellular stress [27]. In some instances stress granule growth will persist after removal of the stressor [28] or will dissolve even before the stress has been removed [29]. Moreover, stress granule clearance in mammalian cells can also occur by autophagy [30]. Stress graules serve as sites of mRNA triage where mRNP complexes are monitored for integrity and composition. Once the stress has been removed, they disassemble and mRNAs can exchange with tRNP granules [21], or be repacked into translationally competent mRNAs and translation can occur [28, 31, 32] or are mRNAs are selectively exported to associated processing bodies for degradation [33]. The primary protein components for stress granules formation include TIA1 cytotoxic granule-associated RNA-binding protein (TIA-1), TIA1 cytotoxic granule-associated RNA-binding protein-like 1 (TIAR), and GTPase Activating Protein (SH3 Domain) Binding Protein 1 (G3BP) along with poly(A)-binding protein (PABP) and the 40S ribosomal subunit [14, 19]. Stress granules also recruit translation initiation complex (i.e., eukaryotic translation initiation factor 2 (eIF2, eiF3, and eiF4E), multiple enzymes and signaling molecules, scaffolding and adaptor proteins, ubiquitin-modifying enzymes, RNA helicases, ribonucleases, ribosyl-, glucosyl- and methyl-transferases (reviewed in [34]). A number of disease-linked proteins are also recruited to stress granules, these proteins include FMRP, SMN, FUS, TDP-43, Ataxin-2 (ATXN2), and other RNA-binding proteins (Table 1) [27, 29, 35–41]. The role RNA-binding proteins have in the assembly and disassembly of stress granules and subsequent effects on protein translation is not fully understood, but it may be that they bring mRNAs to these granules and help protect and repress translation.

**Table 1:** Summary of RNA-binding proteins associated with RNA granules and linked to neurological diseases

RNA-binding protein	RNA granule	Link to disease	References
Angiogenin (ANG)	SG	Mutations in ALS and PD	[37, 98, 99]
Ataxin-2 (ATXN2)	SG	PolyQ expansions in ALS and SCA2	[27, 36, 95, 96]
Ewing sarcoma protein (EWS)	SG	Mutations in ALS, inclusions in FTD	[38, 100, 101]
Fragile X mental retardation protein (FMRP)	tRNP, SG, PB	Mutations in FXS	[19, 27, 44, 59]
Fused in sarcoma (FUS)	tRNP, SG	Mutations and inclusions in ALS, FTD & PQE	[6, 24, 35, 38, 104]
Heterogeneous nuclear ribonuclearprotein (hnRNPA2B1)	SG	Mutations in ALS, FTD and PGD	[39]
Heterogeneous nuclear ribonuclearprotein (hnRNPA1)	SG	Mutations in ALS and PGD	[39]
Survival of motor neuron (SMN)	tRNP, SG	Mutations in ALS and SMA	[19, 27, 40, 92–94]
TATA-binding protein-associated factor 15 (TAF15)	tRNP, SG, PB	Mutations in ALS, inclusions in ALS and FTD	[25, 38, 100, 102, 103]
TAR DNA-binding protein (TDP-43)	tRNP, SG, PB	Mutations in ALS, FTD, inclusions in AD and HD	[7, 22, 29, 105, 106]

*AD* Alzheimer’s disease, *ALS* amyotrophic lateral sclerosis, *FTD* frontotemporal dementia, *FXS* fragile X syndrome, *HD* Huntington’s disease, *PB* processing body, *PD* Parkinson’s disease, *PGD* Paget disease, *PQE* polyQ expansion disease, *tRNP* transport ribonucleoprotein particle granule, *SCA2* spinocerebellar ataxia type 2, *SG* stress granule, *SMA* spinal muscular atrophy

Processing bodies are sites enriched with factors involved in mRNA degradation, mRNA surveillance, translational repression, RNA-mediated silencing, and may also be involved in storage of select RNAs and recycling/modification of decay factors. Many processing bodies exchange rapidly with cytoplasmic proteins and contain only a few stable components. Processing bodies have also been shown to interact with tRNPs [17] and are in physically and functionally associated with stress granules; sharing certain proteins, containing the same species of mRNAs and assemble and disassemble in response to drugs that promote or inhibit polysome disassembly [12, 33]. Due to their dynamic nature it is difficult to identify their exact protein composition and fully understand their function (reviewed in [14]). Whereas stress granules form transiently in response to stress, processing bodies are distinct cytoplasmic silencing foci that are present constitutively and can be enhanced by stress. Like stress granules, processing bodies can be induced by stress [32, 42, 43], are composed of several RNA-binding proteins (Table 1) [22, 25, 44] and contain translationally stalled mRNAs that can be targeted for degradation or may return to translation [32, 42, 43, 45]. However, processing bodies can be distinguished from stress granules by the presence of the RNA-binding proteins, decapping mRNA 2 (DCP2), decapping enzyme 1a (DCP1A), and U6 Small Nuclear RNA-Associated (LSM), trinucleotide repeat-containing 6A (TNRC6A also know as GW182) proteins [19, 46]. Moreover, due to their close ties with mRNA degradation, processing bodies also contain proteins involved in mRNA decay (i.e., decapping factors, DCP1/DCP1), RNA degradation (i.e., deadenylase complex CCR4/CAF1/NOT), nonsense-mediated mRNA decay proteins, ARE-mediated decay factors, and RNAi machinery (GW182 and Argonautes) (reviewed in [14]).

## Mechanisms of local translational regulation by RNA-binding proteins

mRNP granules form in the nucleus. When not being actively translated, cytoplasmic mRNPs can assemble into large multi-mRNP complexes (i.e., tRNP granules) or be permanently disassemble and degraded (reviewed in [47]). The transportation of mRNAs to dendrites by tRNP granules is thought to occur in a translationally dormant state. Consistent with this model, eukaryotic translation initiation factor 4AIII (eIF4AIII), a protein involved in pre-mRNA splicing in the nucleus, was shown to be associated with dendritic mRNA [48]. Because eIF4AIII would be removed from the mRNA by the first ribosome to read the transcript, this suggests that these dendritic mRNAs have not been previously translated. The mechanism by which tRNP granule protein components can selectively inhibit mRNA translation until the proper cues occur is not known. In the case of mammalian Staufen-1 and -2 proteins, some tRNP granules contain ribosomes, whereas others do not. When fractionated by size, the largest Staufen pools contained ribosomes and endoplasmic reticulum, whereas the smaller tRNP granules were cofractioned with kinesin and were free of ribosomes and endoplasmic reticulum [49]. This evidence suggests that the smaller tRNP granules might represent the translationally repressed pool of this type of RNA granule. Moreover, in response to neuronal activity, mRNAs can be released from tRNP granules to the polyribosome fraction where transcripts are actively translated [50]. However, repression of translation by tRNP granules has also been reported in response to neuronal activity (discussed below). The determining factor between active or repressed translation may be controlled by the protein composition of the granule.

The role of the protein components of tRNP granules is an ongoing question. There is evidence to suggest that RNA-binding proteins which associate with tRNP granules have the ability to regulate mRNA repression and/or translation. The RNA-binding protein, ZBP1 associates with and transports β-actin mRNA to synapses [51]. Once in the cytoplasm ZBP1 can be dissociated from the mRNA by Src phosphorylation, allowing synthesis of β-actin, which is necessary for cell migration and neurite outgrowth [52]. ZBP1 can repress the joining of ribosomal subunits in the cytoplasm, thereby regulating translation initiation [52]. Another regulator of mRNA translation in neurons is the CPEB family of RNA-binding proteins. In this example, CPEB1 functions as both a repressor and activator of translation. Initially, CPEB1 binds near the end of the 3′UTR anchors a complex of proteins that include an eIF4E-binding protein (maskin), a poly(A)-polymerase (Gld2), a scaffolding protein (symplekin), and a deadenylase (poly(A)-specific ribonuclease (PARN) [53–55]. In oocytes, binding of CPEB1 to the mRNA initially inhibits mRNA translation through the interaction of an maskin and eIF4E; however, CPEB1 phosphorylation leads to the dissociation of PARN from the complex and subsequent polyadenylation of the 3′ tail by Gld2 [55]. This polyadenylation results in the dissociation of maskin from eIF4E and the activation of translation [56]. Neurons likely use a similar process to regulate translation in dendrites [57, 58].

One well-known regulator of translation is the RNA-binding protein FMRP, which regulates translation and mRNA transportation to dendrites. Mutations in the gene encoding FMRP are associated with a loss of function and cause one of the most common inherited forms of autism, Fragile X syndrome [59]. FMRP-associated tRNP granules traffic into dendrites upon activation of group 1 metabotropic glutamate receptors (mGluR) to regulate translation [60]. The binding of FMRP to mRNA is shown to inhibit translation [61]; however, mice that lack FMRP exhibit both up- and down-regulation of FMRP mRNA targets [62, 63]. Moreover, in the absence of FMRP, the over translation of mRNA normally regulated by FMRP in the dendritic spine leads to excess internalization of the *α*-amino-3-hydroxy-5-methyl-4-isoxazolepropionic acid (AMPA) receptor and enhanced long-term depression following mGluR activation [64]. Consistent with this multifaceted role in mRNA translation, FMRP can associate with multiple types of RNA granules that contain both actively translating polyribosomes [65, 66] and non-translating RNPs [67, 68]. At the synapse, a model for FMRP function has been proposed whereby mGluR activation results in a disinhibition of FMRP-bound mRNA. Where in acute mGluR stimulation, FMRP is dephosphorylated by protein phosphatase-2A (PP2A), ubiquitinated and degraded, which relieves translation inhibition and enable immediate translation of FMRP-bound mRNAs [60, 69]. In contrast, extended activation of mGluR1 (1 min or more) results in rapamycin (mTOR)-mediated PP2A suppression and FMRP rephosphorylation, which coincides with translation inhibition of select FMRP target transcripts [69].

Several studies indicate that FMRP plays a critical role in regulation of mRNA translation by serving as a link between transport tRNP granules and polyribosomes. This is based on the distribution of FMRP to these fractions. For instance, FMRP is present in polyribosomes and acts to stall ribosomal translocation during elongation of its target mRNAs [61]. The phosphorylated form of FMRP associates with stalled polyribosomes, whereas unphosphorylated FMRP associates with actively translating polyribosomes [70]. Presumably, dephosphorylated FMRP no longer acts as a repressor of translation, allowing the ribosomes to translate mRNA and run-off. FMRP can also regulate translation through an association between miRNAs and the RNA-induced silencing complex (RISC) [71]. FMRP regulates translation by acting on the RISC complex containing miR-125a to modulate translation of postsynaptic density protein 95 (PSD-95) [72]. When FMRP is phosphorylated, FMRP recruits Argonaute 2 (Ago2) complexes containing miR-125a and represses synthesis of proteins, such as PSD-95. In response to mGluR signaling, FMRP dephosphorylation leads to the release of RISC from PSD-95 mRNA, which stimulates translation [72]. In this case, FMRP acts as a bridge to deliver miRNA to complementary mRNAs. Thus, dysregulation of microRNAs is also part of how RNA-binding proteins exert translational control, a potential process that is disrupted in fragile X and diseases involving other RNA-binding proteins. We have only mentioned some of the findings for FMRP function and regulation, for more detailed review see [73]; however, the mentioned body of work highlights the complexity of RNA-binding proteins in regulating translation in the dendrites. Knowing the dynamic nature of RNA granules, the existence of different pools of tRNP granules, and the multi-protein complexes that make up these granules at any given moment, it is still unclear how RNA-binding proteins collectively influence translation, the transition of targeting mRNAs to polyribosomes, and subsequent regulation of translation.

## Dendrite and synapse attrition and the link to neurodegenerative disorders

Neuron dendritic branching, synapse formation, and stabilization play significant roles in the structural and functional plasticity of the brain. Precise synapse development and formation is important for accurate neuronal network activity and normal brain function. Therefore, it is not surprising that alterations in dendrite morphology or defects in neuronal development, including changes in dendrite branching patterns, fragmentation of dendrites, retraction or loss of dendrite branching, and changes in spine morphology and number, contribute to disease. In particular, these changes have been observed in several neurodegenerative, neurodevelopmental, and neuropsychiatric disorders, such as, ALS [74], FTD [75], Alzheimer’s disease, Down’s syndrome, autism spectrum disorders, fragile X syndrome, Rett syndrome [76], anxiety and depression [77, 78], schizophrenia [79], and Parkinson’s disease [80]. Various studies report that many neuropsychiatric disorders are characterized by dendritic and synaptic pathology, including abnormal spine density and morphology, synapse loss, and aberrant synaptic signaling and plasticity [81, 82].

Animal models of neurodegenerative diseases also show changes in dendritic branches and abnormal spine morphology including animal models of ALS [83, 84] and FTD [75, 83], as well as in models of mental retardation and fragile X syndrome [85, 86]. In particular, there are several examples of RNA-binding proteins which have been found to affect neuronal morphology and function, and their deficiency are implicated in causing alterations in dendritic branching and spines which underlie the associated neurological diseases. Alterations in dendritic spines in fragile X syndrome and the corresponding FMRP knockout mouse model is characterized by an excess of long and thin filopodial-like spines and a reduction in mature spines [86], which is likely due to dysregulated protein synthesis at synapses. Staufen associates with tRNP granules along with FMRP, TDP-43 and huntingtin [22, 87, 88] and may indirectly contribute to neurological diseases. This is predicted to occur in diseases related to FMRP, TDP-43 and huntingtin where RNA granule biology is affected, which also alters Staufen localization and function. The relevance of Staufens and disease comes from its role in maintaining dendrites and spines. For instance, in hippocampal neurons derived from Staufen-1 knockout mice have deficits in dendritic delivery of β-actin tRNP granules and these neurons have significantly reduced dendritic tree and develop fewer synapses [89]. Staufen-2 is shown to be essential to dendritic spines in mammalian hippocampal neurons, wherein neurons deficient for Staufen-2 have reduced dendritic spines and increased filopodia, which are caused, in part, from impaired β-actin mRNA localization [90]. FUS, which will be discussed in more detail below, is locally translated and localized to spines in response to mGluR5 activation [83, 91]. Neurons cultured from FUS-null mice showed an excess of filopodial-like or thin spines lacking heads and a reduction of mature spines having a mushroom shape [91] and transgenic mice expressing ALS-FUS-associated mutations also have fewer mature spines and reduced dendritic branches [83, 84]. Collectively, these studies demonstrate the role of mRNA regulation by RNA-binding proteins in translation affecting spine development, which likely have important consequences for synaptic plasticity, learning, and memory [10]. There are diverse mechanisms and genetics that underlie the loss of dendritic branching patterns and synapses for these disorders. However, protein translation at synapses has emerged as a central process in the maintenance of dendritic branches and synapses. Thus, altered RNA metabolism at synapses may be the root cause of neurodegeneration observed in ALS and FTD.

## RNA-binding proteins associated with neurodegenerative disorders

In the last 10 years more RNA-binding proteins have been identified through genetic studies to be linked with causing neurodegenerative diseases. Genes that encode SMN [92–94], ATXN2 [95, 96], senataxin (ALS4) [97], angiogenin (ANG) [98, 99], ewing sarcoma protein (EWS) [100, 101], heterogeneous nuclear ribonucleoproteins (hnRNPA2B1 and hnRNPA1) [39], TATA-binding protein-associated factor 15 (TAF15) [100, 102, 103] along with previously discussed FUS [6] and TDP-43 [7], have functions in post-transcriptional regulation of RNA and mutations in these genes cause motor neuron degeneration and other neurological disorders (Table 1). Additionally, several of these proteins have also been linked with neuropathology of diseases (Table 1) [6, 7, 100, 104–106]. Mutations in the RNA-binding proteins, FUS [6], and TDP-43 [7] are identified as genetic causes of both ALS and FTD. The role in how these proteins contribute to disease is not fully understood, but dysregulation of RNA metabolism as in fragile X syndrome is likely a major factor in the contribution to neurodegeneration. Here, we will discuss the prominent ALS and FTD-associated RNA-binding proteins, FUS, and TDP-43, and their roles in promoting neurodegeneration, potentially through altered protein translational regulation at synapses.

### FUS properties and function

FUS is part of the FET family of RNA-binding proteins that include EWSR1 and TAF15 [107]. FUS binds to thousands of cellular RNAs [108–111] through its two RNA recognition motifs (RRMs), zinc-finger domain, and three arginine-glycine-glycine (RGG) boxes [112–114]. FUS exists in different ribonucleoprotein complexes involved in pre-mRNA splicing, mRNA stability and mRNA transport [115–118] and miRNA biogenesis [119]. FUS co-purifies with the spliceosome [120, 121], different pre-mRNA splicing [117, 118] and miRNA Drosha complexes [122]. At its steady state, FUS is localized to the nucleus of cells and in nuclear gemini of coiled bodies (gems) along with TDP-43 and SMN, where all three proteins function in spliceosome maintenance [115]. A feature of FUS is that it binds the whole length of nascent RNA [109, 111], which implies its close association with transcripts from the time of production and supports the idea that it may be involved in transcriptional elongation. This is further supported by the finding that FUS has a close association with the C-terminal domain of RNA polymerase II and its association with that complex can influence phosphorylation and transcriptional activation [123, 124]. FUS binds to pre-RNA and mRNA at introns, coding sequences, 3′UTR and 5′UTRs and also targets noncoding RNAs [108, 109, 111]. The impact of RNA regulation by FUS is demonstrated in FUS-null cells where there is a broad misregulation of RNA processing involving mRNA regulation and pre-mRNA splicing [109, 111]. Functional classification of FUS RNA targets reveals a number of essential cellular processes. Notably, a significant number of their RNA targets encode proteins that function at the synapse, several of which are involved in neuronal development and synaptic transmission [108, 109, 111].

The majority of FUS is localized to the nucleus of cells; however, it can localize to different cellular compartments and RNA granules in response to various stimuli. This is facilitated by its non-classical proline-tyrosine nuclear localization sequence (PY-NLS) and nuclear export sequence (NES) [125]. For example, treatment of acute cortical slices or hippocampal neurons with mGluR1/5 agonists, results in local translation [83], and localization to synapses [91]. FUS has also been found in tRNP granules localized to dendrites [23, 24] and localized to synapses where some of FUS associates with the NMDA receptor [24]. Although it has not been tested, the role of FUS in the spines may control localization, anchoring, or regulating mRNAs at the synapse. The function of FUS in tRNP granules may be to repress or facilitate translation, this has yet to be fully understood. Recently, FUS has been detected in tRNP granules containing the tumor suppressor protein, adenomatous polyposis coli (APC) in hippocampal neurons, and post-mortem tissues from FTD-FUS patients [126]. In APC-tRNP granules, FUS is demonstrated to be required for the efficient translation of associated transcripts. As discussed above, this is an unexpected finding given that tRNP granules are thought to be translationally dormant. Interestingly, overexpression of ALS-FUS mutations causes the spontaneous formation of APC-tRNP granules which are translationally active [126]. FUS has also been purified from polyribosomes [127], which suggests it has an active role in regulating translation and that it could function in a similar way as FMRP to bridge tRNP granules and translation machinery. In response to oxidative stressors like sorbitol and sodium arsenite, FUS associates with cytoplasmic stress granules [35, 38, 41], which indicates it also has the capability to be an active repressor of translation. Finally, the association of FUS with the Drosha complex and miRNA biogenesis [122], suggests that FUS can repress translation by distinct mechanisms. Future work should identify the relationship between FUS and RNA granule dynamics and the role FUS plays in the regulation of translation.

### FUS and neurodegeneration

The majority of familial ALS mutations that are identified in the gene encoding FUS occur in its C-terminal PY-NLS [128], altering the cytoplasmic localization of the protein and are aggregate prone [35, 129]. Mutations in the 3′UTR of FUS have also been identified, which results in increased FUS expression and cause ALS [130]. The extent to which ALS-FUS mutations localize to the cytoplasm correlates with disease severity [35, 129]. FTD genetic mutations are identified in the RGG1 and N-terminal region, but as demonstrated in cell culture, cytoplasmic localization of FUS is not as prominent [131]. Post-mortem examination of ALS patients with FUS mutations shows abnormal accumulation of FUS in the cytoplasm or nucleus of motor neurons [6]. Similarly, familial FTD and some sporadic forms of the disease have similar FUS aggregation, but in FTD affected brain regions [6].

Animal models expressing various FUS mutations and wild-type FUS reproduce aspects of the human diseases. For instance, *D. melanogaster* [132], *C. elegans* [133], and *R. norvegicus* [134], models that overexpress ALS-FUS mutations causes motor defects in these models, although it is unclear why overexpression of wild-type FUS in these models has little toxicity given that ALS-FUS 3′UTR mutations cause an increase in FUS expression [130]. Transgenic mouse models harboring human wild-type FUS or the ALS-FUS R521G mutation both develop deficits in motor function, motor neuron denervation, and inflammation die before reaching adulthood [83]. However, persistent reduction of dendritic branching and mature spines is only present in the FUSR521G transgenic mice [135], which has also been shown in the transgenic mouse model containing the R521C mutation [84]. These transgenic mouse models of ALS-FUS show similar deficits in dendritic branches and spines as reported in FUS-null hippocampal cultures [91]. These observations suggest that the mechanism of toxicity of these mutants may be due to a partial loss of function regarding RNA regulation and a gain of function regarding the toxicity the mutation causes at the synapse. Meanwhile, a recent study in *D. melanogaster* demonstrates that replacement of the endogenous FUS homolog with the human FUS R521C mutation causes defects in synapse structure and function that precedes motor neurodegeneration [136], which is consistent with the observations in FUSR521G transgenic mice [83]. These observations may be explained by the finding that the FUSR521G mutation is not regulated in the same manner as wild-type in response to neuronal stimulation [83]. It is possible that in response to mGluR, FUS is normally translated at the synapses; however, translation of the FUSR521G protein is dysregulated, which may have negative implications at the synapse. A commonality among all animal models is that they do not develop large amounts of pathological FUS aggregates. This could indicate that FUS aggregation is not central to the disease process, and/or it could suggest that overexpressing FUS causes toxicity by altering its other biological processes as indicated in animal models that have altered dendritic branches and spines.

A potential significance of FUS aggregation comes from the finding that several stress granule markers are also deposited in the aggregates [6]. This has led to several studies that examine stress granule dynamics of FUS mutations, these studies have been more easily modeled in cell culture. In general, ALS-FUS PY-NLS mutations which correlate with disease severity are more prominently localized to the cytoplasm and form larger stress granules in the absence and presence of stress [35, 129]. For instance, recruitment of GFP-FUS into perinuclear stress granules is extensive for truncation mutants R495X or G515X compared with R521G or H517Q [35]. Moreover, there is an indication that FUS mutations may delay the assembly of stress granules [137] and irreversibly sequester a variety of RNA-binding proteins and mRNAs [138]. Despite the close contact of processing bodies with stress granules under conditions of acute stress, GFP-FUS and mutant variants do not incorporate into associated processing bodies or affect docking to stress granules [35]. However, FUS mutations impact the number of processing bodies [137, 138]. The functional implications for these findings are still not clear. It has also been shown that R521G and H517Q mutations have reduced binding to RNA [110]. Not all mutations have been tested for their ability to bind RNA, but the altered ability of the mutants so far tested to bind mRNAs and sequester them in RNA granules may be an important factor in disease pathogenesis. Until now, the model put forth is that FUS mutations somehow “seed” for protein aggregates which sequesters more FUS and other proteins, thus depleting the cell of essential proteins which leads to cell death. This model still needs in vivo data to support the observations made in cell culture.

### TDP-43 properties and function

FUS and TDP-43 have often been reported as having a similar role in disease and biology, but there are some distinctions and similarities, which we will point out in this section. TDP-43 is a member of the heterogeneous nuclear ribonucleoprotein (hnRNP) family of proteins [139]. Like FUS, TDP-43 is a global regulator of gene expression and binds thousands of RNAs [140–142] through its two highly conserved RNA recognition motifs (RRM1 and RRM2), wherein the RRM1 is the major domain for binding RNA and DNA [143, 144]. TDP-43 regulates transcription and multiple aspects of RNA processing and function, including pre-mRNA splicing, mRNA stability, transport, translation [140, 142, 145], and miRNA biogenesis [146, 147]. TDP-43 co-localizes with splicing structures in the nucleus [115], Drosha [122] and DICER complexes [147]. Additionally, TDP-43 interacts with many proteins and RNAs and functions in protein:RNA complexes [142, 148]. TDP-43 is ubiquitously expressed and localizes primarily to the nucleus of cells and can localize to splicing structures known as GEMs [115]. TDP-43 targets thousands of RNAs, which has been shown in vivo and in various model systems [140–142]. Binding RNA through its highly conserved RRMs, TDP-43 has enriched binding to over 4500 RNA species, preferentially localizing to introns, 3′UTR and 5′UTRs and non-coding RNAs [140–142]. Deletion of TDP-43 in cells leads to broad misregulation of mRNA and pre-mRNA splicing [140, 141]. Similar to FUS, TDP-43 targets RNAs of diverse biological importance. In the context of synaptic biology, it shares similar targets to FUS, but with different binding sites [108, 111, 140–142].

TDP-43 can localize to different cellular compartments and RNA granules via a classical NLS and NES [149]. It too is responsive to various stimuli which somehow directs the localization of TDP-43 to these specialized areas. Under basal conditions TDP-43 resides in the dendrites of hippocampal neurons and co-localizes with RNA granules, some of which stain positive for processing bodies, and it co-localizes with the post-synaptic protein, PSD-95. These granules contain RNAs including β-actin and calmodulin kinase II α (CaMKIIα) mRNA. Upon depolarization, TDP-43 co-localizes FMRP and Staufen-1 in tRNP granules within dendrites [22]. In response to oxidative stressors, TDP-43 localizes to the cytoplasm and into stress granules [22, 29, 150–152]. TDP-43 has not been costained with processing bodies in other studies [153]; however, this does not preclude that TDP-43 can associate with these structures under some conditions because many proteins, such as Staufen, FMRP, and HuR are present in processing bodies and stress granules depending on the conditions [154]. TDP-43 has also been found as an integral component of the Dicer complex and is required for the cleavage of pre-miRNAs by Dicer and for the recruitment of Argonaute 2 (Ago2) to the catalytic engine of RISC, to miRNA bound by Dicer [147, 155]. In this study, TDP-43, but not FUS, was found as a component of nuclear Drosha complexes that contain DGCR8, which is indispensable for pri-miRNA processing [146]. TDP-43 is shown to facilitate the production of a subset of precursor miRNAs (pre-miRNAs) by both interacting with the nuclear Drosha complex and binding directly to primary miRNAs (pri-miRNAs) [146]. Furthermore, cytoplasmic TDP-43, which interacts with the Dicer complex, promotes the processing of some of these pre-miRNAs to miRNAs [146]. The implications for TDP-43 involvement in miRNA biogenesis were shown to be indispensable for neuronal outgrowth [146]. Taken together, these findings suggest that the maturation of a subset of miRNAs is modulated at multiple steps by TDP-43, which reveals a unique function of TDP-43 not only in the nucleus but also in the cytoplasm. As demonstrated in this work, TDP-43 is functioning in a similar manner as FMRP in translational repression via miRNA regulation.

### TDP-43 and neurodegeneration

TDP-43 is a major component of ubiquitinated inclusions found in the brain and spinal cord of the majority of ALS patients [156, 157]. In approximately 50 % of FTD patients TDP-43 is found to be the pathological hallmark, the remaining cases are TDP-43 negative and most of which have tau-positive neuropathology [2]. There are now more than 40 TDP-43 familial ALS mutations that have been identified and the majority are found in the C-terminal glycine region [128]. Mutations in the 3′UTR of TDP-43 have also been identified, resulting in increased levels of TDP-43 [158]. Familial FTD mutations in TDP-43 are less prevalent [128]. Unlike FUS, the identified ALS-TDP-43 mutations do not occur in the NLS or NES domains and the impact on localization is not as obvious. Most of the mutations occur in the glycine-rich region, which is shown to mediate protein interactions [159] and contains a predicted prion-like domain [160, 161]. Despite extensive research over the last several years, the pathogenesis of these mutations is still unclear.

Animal models of TDP-43 demonstrate that altering endogenous levels of the wild-type protein or expressing ALS-TDP-43 mutations is highly toxic and reproduces aspects of ALS and FTD [135, 162–166]. Depletion of TDP-43 in *D. melanogaster* results in reduced lifespan and locomotor defects due to alterations in dendritic branching and synapses [167–170], overexpression also caused loss of motor function and is accompanied by a decrease in dendrites and synapses [170, 171]. In *D. rerio*, both overexpression of human TDP-43 and knockdown of *TARDBP* result in swimming behavior defects caused by defective neuronal axon formation, and premature and excessive branching [172]. Similarly, transgenic mouse models of hTDP-43 expressing either wild-type or ALS-associated mutations cause motor defects [162–164]. TDP-43^M337V^ transgenic rats expressing mutant proteins in motor neurons recover their motor function when TDP-43^M337V^ expression is turned off [135]. This study suggests that mutant TDP-43 in motor neurons is sufficient to promote the onset and progression of ALS-like degeneration and that, most importantly, its toxic effects are reversible. TDP-43 knockout mice die between embryonic day E3.5 and E8.5 and TDP-43 null-embryonic stem cells are not viable [165]. Conditional knockout of TDP-43 in motor neurons exhibit progressive development of ALS-related motor phenotypes and accumulation of ubiquitinated proteins [166]. Although dendrite and spine morphology have not been extensively studies in murine models, knockdown or overexpression of ALS-TDP-43 mutant proteins (A315T, Q331K, and M337V) in cortical neurons have been shown to cause both abnormal neurites and decreased cell viability [173]. Depletion of TDP-43 leads to an increase number of mature spines in hippocampal neurons, with an increase clustering of AMPA receptors on the dendritic surface which corresponds with increased neuron firing [174]. Overexpression of TDP-43 causes a loss of mature spines [174]. In knockdown cells, these changes correlate with increased level of Rac1 [174], a positive regulator of spinogenesis, suggesting that TDP-43 may be an upstream suppressor of Rac1 translation. These models indicate that the balance of TDP-43 levels is important for normal biological processes, at least in the case of the loss of TDP-43 and given the large number of RNA targets of this protein we can predict that there would be a significant biological impact. However, in the case of overexpression of either wild-type or TDP-43 mutations, the gain of function mechanism is not clear.

Another property of ALS-TDP-43 mutations and wild-type TDP-43 is that they are actively recruited to cytoplasmic stress granules in response to stress [150]. Moreover, TDP-43 ALS-associated mutations (i.e., A315T, G348C) are more sensitive to oxidative stress, and form larger stress granules in the cytoplasm, but do not form stress granules in the absence of stress [29]. The region in which these mutations are located appears to be important for stress granule recruitment because the deletion of the C-terminal glycine-rich domain abolishes TDP-43’s association with these granules [29, 113, 152]. In addition to this, the N-terminal RRM1 is also necessary for TDP-43’s incorporation into stress granules [113]. Together this indicates that stress granule recruitment of TDP-43 requires both RNA-binding and protein–protein interactions. Other consequences of TDP-43 mutations include increased half-life of the protein [116] and the stability of its target mRNAs [175]. As in the case of FUS, TDP-43 and stress granule markers co-stain in ALS and FTLD-U pathological aggregates [113], which suggests that TDP-43 may also “seed” stress granules formation in pathological aggregates [150].

Much of the field has focused on stress granules; however, TDP-43 mutations have been shown to affect tRNP granule formation and migration. TDP-43 is associated with RNA granules that are prevalent throughout the dendritic arbor in neurons. “Aggregation” of TDP-43 is also important for the formation of these neuronal tRNP granules, and it is reasonable to assume disease-linked mutations might alter granule formation. Indeed, ALS-TDP-43 mutations are shown to increase the size of neuronal TDP-43 granules in the dendritic arbor of rat hippocampal neurons under basal conditions [153]. Depolarization of rat hippocampal neurons with KCl stimulates TDP-43 granule migration into dendrites, but A315T and Q343R TDP-43 granules migrate shorter distances and into fewer dendrites than wild-type TDP-43 [153]. The mutations correspondingly reduce the granule density, movement, and mobility of TDP-43 granules. Interestingly, some TDP-43-positive RNA granules also exhibit a close interaction with processing bodies [153]. In another study that examined TDP-43 tRNP granules, TDP-43 mutations impair mRNA transport in stem cell-derived motor neurons from ALS patients bearing any one of three different TDP-43 ALS causing mutations [176]. These findings highlight novel elements of TDP-43 biology that are affected by disease-linked mutations and suggest a neuronally selective mechanism through which TDP-43 mutations might elicit neuronal dysfunction. The functional implications could be the absence of important RNAs to sites of local translation or misregulated mRNA products. Given the close association of TDP-43 with tRNP granules, the functional implications of TDP-43 mutations in tRNP granule formation and trafficking will also need to be examined.

## Future perspectives

The role of RNA-binding proteins in translational control and how it relates to neurodegeneration is gaining interest in the field. This has occurred for a number of reasons: (1) identification of genetic mutations in genes encoding proteins that are involved in RNA regulation and linked to neurodegenerative diseases; (2) in vivo disease models do not recapitulate pathological aggregate phenotypes of the diseases and in vitro stress granule models do not fully explain the global impact on RNA misregulation; (3) at least in the case of TDP-43-FTD, cross-linking and immunoprecipitation of TDP-43 and transcriptome analysis reveal very few changes in RNA-binding profiles from control patients [141], and the genes that are altered do not link directly to causes of neurodegeneration; (4) synaptic dysfunction precedes neurodegeneration and recent evidence shows that tRNP granule formation, localization and dynamics are affected by disease mutations of RNA-binding proteins. This suggests that mRNA transportation, processing and translation may be affected, which may lead to defects at the synapse and trigger the earliest events during the neurodegeneration process (Fig. 1b).

If alterations of RNA granule dynamics, transport, and translation are important factors in maintaining the synapse, then how are RNA-binding proteins coordinating these processes? Dynamic exchange of mRNA between tRNP granules, stress granules and processing bodies is in part coordinated by specific RNA-binding proteins. There is evidence that supports RNA-binding proteins are essential in the shuttling of mRNA to and from these specialized granules until such time that the mRNA is translated in polyribosomes. The impact of deletion of RNA-binding proteins can lead to alterations in dendritic branches and spines as well as impact translation. Key questions that arise are: How do RNA-binding proteins target mRNA to each RNA granule? What cues initiate mRNA exchange between RNA granules? What post-translational modifications do RNA-binding proteins undergo to coordinate exchanges between RNA granules? It is clear that stress granule formation and the presence and numbers of processing bodies are affected by ALS-FUS and ALS-TDP-43 mutations. Recent work shows that tRNP granules are also affected by ALS-FUS and ALS-TDP-43 mutations. Putting all this evidence together would strongly suggest that mutations at least in these RNA-binding proteins would affect the dynamics of mRNP–RNA granule exchanges, which we defined here as the transfer of mRNA:Protein complexes between RNA granules in response to cellular stimuli and cues (Fig. 1b). More concerted efforts need to be made to examine the effects on local translation and the downstream consequences at the synapse.

Finally, if RNA-binding proteins affect mRNP–RNA granule exchange, how does this affect local translation and synaptic function, and can the resulting effects lead to neurodegeneration? The strongest precedence by an RNA-binding protein has been set for FMRP in the autism disorder fragile X syndrome. In this case, loss of FMRP causes changes in synaptic morphology and function in fragile X syndrome, but this disease does not involve neurodegeneration. The challenge in the neurodegeneration field will be to mechanistically link dysfunction of mRNP-RNA granule exchange and local translation with initiation of neurodegeneration. This will be difficult to dissect given that RNA-binding proteins like FUS and TDP-43 regulate thousands of RNAs in both the nucleus and cytoplasm. A particular focus will need to be on target RNAs that encode proteins critical for synaptic function at both the steady state and in response to neuron stimulation. As observed in the FUSR521G transgenic model, defects in translation are more prominent at synapses in response to mGluR stimulation. The function of FUS and TDP-43 at synapses is not well understood, but the existing evidence point to a prominent role in maintaining the function and integrity of synapses: these RNA-binding proteins have been purified with synaptic tRNP granules; their expression is elevated upon neuron stimulation; genetic deletion results in altered dendritic spines and branches in primary cultured neurons. This in addition to their genetic link to ALS and FTD strongly implicate RNA-binding proteins as having a major role in causing alterations in RNA metabolism locally at the synapse, which would alter synaptic function and trigger neurodegeneration.

## Abbreviations

ALS: Amyotrophic lateral sclerosis
AMPA: *α*-Amino-3-hydroxy-5-methyl-4-isoxazolepropionic acid
CPEB: Cytoplasmic polyadenylation element-binding protein
FTD: Frontotemporal dementia
FMRP: Fragile X mental retardation protein 1
FUS: Fused in sarcoma
hnRNP: Heterogeneous nuclear ribonucleoprotein
mGluR: Metabotropic glutamate receptor
NES: Nuclear export signal
NLS: Nuclear localization signal
PSD-95: Postsynaptic density protein 95
PY-NLS: Proline-tyrosine nuclear localization signal
RISC: RNA-induced silencing complex
RGG: Arginine-glycine-glycine
RNP: Ribonucleoprotein particles
RRM: RNA recognition motif
TDP-43: Transactive response DNA-binding protein 43
tRNP: Transport ribonucleoprotein particles
UTR: Untranslated region
ZBP1: Zipcode-binding protein 1
